# ‘I felt pain. Deep pain…’: Experiences of primary caregivers of stroke survivors with aphasia in a South African township

**DOI:** 10.4102/ajod.v7i0.368

**Published:** 2018-03-08

**Authors:** Khetsiwe P. Masuku, Munyane Mophosho, Muziwakhe Tshabalala

**Affiliations:** 1 Department of Speech Therapy and Audiology, University of the Witwatersrand, South Africa; 2 Department of Physiotherapy, Sefako Makgatho Health Sciences University, South Africa

## Abstract

**Background:**

Aphasia is an acquired impairment in language and in the cognitive processes that underlie language. Aphasia affects the quality of life of the person with aphasia (PWA) and his or her families in various ways in diverse contexts and cultures. It is therefore important that speech language therapists understand how different contextual and cultural factors may mediate experiences.

**Purpose:**

The aim of the study was to describe the caregiving experience of female caregivers of PWA residing in Tembisa, a township situated in the east of Johannesburg.

**Method:**

Qualitative, semi-structured interviews were conducted with primary caregivers of PWA. Purposive sampling was used to recruit 14 primary caregivers of PWA who were daughters, daughters-in-law or wives of the PWA. The interviews were conducted in participants’ first language and analysed by the researcher, who is proficient in isiZulu. Data were analysed according to the principles of thematic analysis.

**Results:**

Findings indicated that caregivers are unfamiliar with aphasia and the support available to them. Participants experienced frustration and found communication to be challenging owing to their lack of communication strategies. The participants’ experiences reflected their context-specific experiences, such as feminisation of caregiving, barriers to healthcare, the influence of low health literacy and contextual perspectives on stroke and aphasia.

**Conclusions:**

Contextual factors of caregivers in Tembisa have an influence on the experiences between caregivers and PWA, the feelings of individuals and families and health-seeking behaviours of individuals and families.

## Introduction

The incidence of stroke has risen owing to the disease profile in developing countries, which includes the impact of HIV and AIDS in Africa, the impact of urbanisation on diet and lifestyle (Coovadia et al. 2009) and the high rates of interpersonal violence and motor vehicle accidents (Jamieson, Harrison & Berry 2008). There is a rapidly increasing burden of non-communicable diseases in poor and disadvantaged populations contributing to widening health gaps (Penn & Armstrong 2017: 566). Hence, those living in impoverished and marginalised communities are particularly susceptible to chronic, non-communicable diseases (Sambo & Kirigia 2014).

Stroke remains one of the most devastating of all the non-communicable and neurological diseases, often causing death or gross physical impairment or disability (Mukherjee & Patil 2011). Stroke also results in aphasia, which is an acquired communication disorder caused by brain damage and characterised by impairment of language modalities such as speaking, listening, reading and writing (Hallowell & Chapey 2008).

Aphasia affects caregivers, often in profound ways (Legg 2010), as family members generally become caregivers when a relative sustains disabling conditions as a result of a stroke (Kniepman & Cupler 2014). A ‘primary caregiver of people with aphasia (PCPWA)’ in this study is defined as a family member tasked with providing unpaid care for another family member affected by aphasia as a result of a stroke. Primary caregivers of people with a stroke are generally responsible for facilitating participation of the person with a stroke in his or her daily functional activities (Hilton 2011).

There is a plethora of research that has documented the experiences of caregivers for PWA, as well as their experiences when communicating with the PWA (Earnes et al. 2008; Gillespie, Murphy & Place 2010; Patricio, Jesus & Cruice 2013). These studies have suggested that PCPWAs lack an understanding of what aphasia is and are therefore in need of empowerment through education. There has also been documented findings of how ill-prepared caregivers are regarding their new roles and how to deal with the sudden and often permanent changes caused by aphasia (Avent et al. 2005; Howe et al. 2012). Hence, the need for support in this new role should be emphasised (Howe et al. 2012; Le Dorze & Signori 2010). The changes in the communication environments between partners have been documented and are seen to result in increased emotional reactions, including frustration, strain, stress, anger, burnout and resentment, as well as interpersonal relationship changes such as reduced marital quality of life, limitations in family roles and responsibilities, and restricted social life (Avent et al. 2005; Howe et al. 2012). Primary caregivers of people with aphasia need to be able to communicate and maintain their relationship with the PWA (Howe et al. 2012). Furthermore, they need to be given information on the disorder so that they have a better understanding of what it is all about (Howe et al. 2012; Le Dorze & Signori 2010).

Research in South Africa that has stressed the importance of the influence of context and culture, especially in poverty-stricken communities, in understanding aphasia and informing health-seeking behaviours include studies by Legg (2010), Legg and Penn (2013a, 2013b, 2014) and Penn (2014).

In the African context, women are the main caregivers of persons with disabilities, including aphasia (Chitayat 2009; Cordier 2014; Posner 2016). Two-thirds of the caregivers in South African households are women and this is often accepted as the regular mandate in the South African culture (Chitayat 2009). The feminisation of caregiving and its relations to poverty has been a feature of most caregivers in the developing world (World Health Organization 2002). It is imperative to highlight the implications of leaving caregiving exclusively to women (Kang’ethe 2013). Women are placed into the caregiving role which results in fewer opportunities for socio-economic growth and employment (Makiwane & Berry 2013). The loss of income within the family of a person with a disability will therefore result in poverty (Cordier 2014; Makiwane & Berry 2013), especially if the person with the disability or the PCPWA was the sole breadwinner. Households that have family members with disabilities have particular economic demands on them which other households do not (World Health Organization/World Bank 2011). Besides caregiving, women generally hold more household duties and responsibilities, limiting their opportunities for income generation, and further contributing to their state of poverty (Cordier 2014; Makiwane & Berry 2013).

Feminisation of caregiving has been found to be exacerbated by additional stressors in many South African families (Coetzee 2016), which include exhaustion and burnout, which are often the result of a lack of a break from caring (Mathye & Ekseen, 2015; Sandy, Kgole & Mavundla 2013), compounded by the extreme loneliness experienced by caregivers as a result of not being supported, especially by families within their caring role (Geiger 2012; Mathye & Eksteen 2015). This often results in caregivers feeling isolated from their communities (Mhaule & Ntswane-Lebang 2009).

There are numerous ways in which people with aphasia and their families explain illness and disability in South Africa. Explanations include witchcraft, ancestor reprisal, social causes and ignorance (Legg & Penn 2013a). Some of these explanations can be attributed to poor health literacy, especially in low socio-economic contexts in South Africa. Public awareness and knowledge of aphasia and its effects are limited in comparison with other disorders with similar or lower prevalence (Sherratt 2011). Health literacy is important for informal caregivers because they have the responsibility of health decision-making, communicating with healthcare providers and problem-solving on behalf of the person with a disability (DuBenske et al. 2010).

Many aspects of caregivers’ personal and environmental contexts interact to inform the ways in which they experience caregiving (Bingham 2017); therefore, the current study aimed to highlight the experiences of South African women caregivers of PWAs, who reside in Tembisa, a township situated in Ekurhuleni in the east of Johannesburg. Tembisa was established in 1957, when the segregation policies of the apartheid regime enforced the resettlement of Black South Africans from Alexandra and other areas in Edenvale, Kempton Park, Midrand and Germiston. Tembisa remains a location where the vast majority of residents are Black people (Ekurhuleni Metropolitan Municipality, 2001). People in townships live in a context of poverty, inadequate service delivery, poor housing, high rates of crime and unemployment, and dissatisfaction with the government (Pieterse 2011).

## Methods

### Objectives

The main aim of this study was to provide an in-depth description and discussion of the experiences of PCPWAs who reside in a South African township. In the study, we explored caring for a PWA and we also sought to understand the unique experiences of being a PCPWA in a township context.

### Research design

The study employed a qualitative research design. Semi-structured interviews were conducted with the primary caregivers of PWAs who were recruited through purposive sampling. Purposive sampling is widely used in qualitative research for the identification and selection of information-rich cases related to the phenomenon of interest (Palinkas et al. 2015). The researcher conducted the interviews in isiZulu and audio recorded, transcribed and translated them into English. Translation from isiZulu to English was done for the understanding of the analysis by a wider audience. Throughout the data collection period, interviews were held at convenient times for participants. The researcher also ensured that participants felt comfortable to have a conversation about their experiences (Boyce & Neale 2006). The participants were more likely to have been comfortable communicating with the researcher who comes from the same cultural and linguistic background as they did.

### Participants

A total of 14 PCPWAs took part in the study and were closely related to the PWAs. The mean age of the PCPWA was 38 years – the youngest was 21 years old and the eldest was 65 years old. The older participants were more likely wives (six), and the younger were (five/one) daughters/daughters-in-law of the PWA. The education level of the PCPWA ranged from mostly no formal education to one participant with a university degree. Eight of the participants had been unemployed at the time of the stroke, and six had had to give up their employment so as to become full-time caregivers. All caregivers were women, which is a trend in the South African context (Posner 2016). The experience of caregiving ranged from 3 months to more than 3 years, with nine of the caregivers having cared for the PWA for less than 1 year, three under 2 years and two for more than 2 years (Table 1).

**TABLE 1:** Research participants: Demographic details.

Participant	Age	Duration as a caregiver	Relationship to PWA	Level of education	Occupation before caregiving
1	21 years	9 months	Daughter	Secondary school education	Unemployed
2	23 years	6 months	Daughter	Diploma	Unemployed
3	28 years	6 months	Daughter	Degree	Full time employed
4	33 years	3 months	Daughter-in-law	Diploma	Full time employed
5	34 years	3 years and 3 months	Daughter	Secondary school education	Self employed
6	35 years	6 months	Daughter	Diploma	Full time employed
7	35 years	1 year and 3 months	Wife	Secondary school education	Unemployed
8	37 years	8 months	Wife	No formal education	Self employed
9	37 years	4 months	Wife	No formal education	Unemployed
10	40 years	2 years and 2 months	Wife	Primary school education	Unemployed
11	40 years	1 year	Sister	Secondary school education	Self employed
12	47 years	3 months	Sister	No formal education	Unemployed
13	57 years	1 year and 3 months	Wife	No formal education	Unemployed
14	65 years	2 years	Wife	No formal education	Unemployed

PWA, person with aphasia.

## Materials and methods

A self-developed interview schedule was used to collect data on the experiences of PCPWA. The interview schedule comprised five sections. Section A consisted of demographic information. Section B aimed to extract information on the participants’ understanding of aphasia and its causes. Section C drew information on the participants’ experience of communicating with the PWA. Section D explored the PCPWA’s experiences of caring for the PWA. Section E encompassed questions aimed at describing the kinds of support structures available for PCPWA. Interview questions were developed based on existing literature on the experiences of PCPWAs. The interview guide was developed in English and translated into isiZulu by the researcher and back translated into English by an independent translator to ensure validity. All interviews were audio recorded.

### Data collection procedure

Permission to conduct the study was obtained from the chief executive officer of the tertiary hospital at which the study was conducted. The Speech Therapy and Audiology department of the hospital assisted with the recruitment process by providing the researcher with the contact details of the prospective participants. The researcher made contact with the participants telephonically to request their participation in the study. The hospital provided the researcher with a private room in the Speech Therapy and Audiology department, which is where all the interviews were conducted. Each interview lasted an average of 40 min. Written informed consent for the interviews and audio recordings was obtained from participants in both isiZulu and English. Confidentiality, voluntary participation and disclosure of potential risks and benefits were communicated to the participants before the commencement of the data collection process.

### Data analysis

Data were analysed according to the principles of thematic analysis (Braun & Clarke 2006). The researcher was guided by Shields and Twycross’s (2003) recommendations of what the process of data analysis entails. The first stage of thematic analysis was the verbatim transcription of the isiZulu interviews, which were then translated to English and back translated to isiZulu by an independent coder. This also involved checking for any omissions or errors by the researcher. Secondly, the process included reviewing the translated transcripts several times, while listing emerging and re-emerging themes based on the experiences of the participants. The interviews were collected over 14 weeks; consequently, analysis began while other interviews were going on as per Cresswell’s (2007) guidelines. The coding was done based on the prevalence of the themed pattern in the data set. Prevalence was determined in terms of the number of responses across the entire data set. Themed patterns repeated more than twice by different primary caregivers were considered. Consensus coding was used to identify codes and themes related to caregivers’ experiences. Thereafter, the themes emerged from the transcripts through words, phrases, sentences and paragraphs that represent or symbolise issues relating to experiences of the participants.

### Rigour

Strategies proposed by Lincoln and Guba (1999) were employed to ensure rigour in the study. Credibility was ensured through describing the data collection and data analysis process. Credibility was also ensured through providing verbatim transcriptions of the recorded data including back translation of isiZulu to English. To ensure confirmability, data analysis results were well grounded in data. There were also peer debrief discussions between authors during data analysis to ensure that there was agreement on codes and themes. Detailed field notes were collected during data collection. The researcher spent time prior to the actual research to familiarise herself with the context and research tools prior to a pilot study. Description of the research setting, participants and research processes was given. This is to ensure that the study can be replicated.

### Ethical considerations

Ethical clearance was obtained from the MEDUNSA Research and Ethics Committee (project number: MREC/H/2011:PG).

## Results

From the analysis of the data, six main themes emerged: Understanding of aphasia; Engagement; Support; Emotions: Burnout and burden of care; Challenges to care; and Feminisation of caregiving.

### Understanding of aphasia

Primary caregivers in this study were able to recognise a stroke, although they had only limited understanding of aphasia. They attributed the causes of a stroke to different factors which included witchcraft (the belief that someone bewitched them because of jealousy at work), falls, biomedical rationales (the belief that the person had a stroke, but appeasing ancestors would help them heal), lifestyle aspects (poor diet, smoking and defaulting on hypertension medication), antiretroviral medication and old age. Aphasia was also explained as the PWA having a problem with his or her mind, as can be seen in the following quote:

‘I know that he can’t talk. What I know is how sometimes his mind is going down. He now thinks like a child and cannot talk. He uses his hands. He is behaving like a child. Ja, his mind is just upside down and I am told it’s because of a stroke. I cannot understand how it’s because of a stroke.’ (Participant 8, 37 years old, wife)

Some caregivers did not know what the cause of aphasia was and did not believe that a stroke could result in communication difficulties.

‘No, I don’t want to lie, I really don’t know. All I know is that it is not possible for someone to all of a sudden stop talking. It is just not possible, hmmm, hmmm. It is not possible. You see my child, there are a lot of people with that (a stroke) that I know of and they talk so it is just not possible. That is the reason why I did not say anything about him not speaking to the nurses.’ (Participant 13, 53 years old, wife)

Stroke being attributed to biomedical causes was mostly common in participants who had higher levels of education, as illustrated in the following quote:

‘Well, there are certain causes depicted of them, it’s that it may have been caused by alcohol intake, cigarette intake and he used to be actively doing such things and he is also old, and I mean we don’t really know why he is not talking. I mean, it’s a right-hand stroke, it’s probably the most dangerous, but we don’t know. So it could have been due to multiple factors, I don’t know.’ (Participant 6, 35 years old, daughter; highest level of education is a diploma).

### Engagement

Interactions and family relationships had been affected as a result of the communication difficulties experienced by the PWA. It became clear that the PCPWA did not have the necessary strategies to facilitate communication, even when PWAs could initiate conversations. As conversational strategies had not been taught to the caregivers, to the PWA or to their family, participants experienced communication to be tedious, frustrating and humiliating, and caregivers then tend to lose patience.

‘Okay now honestly, I find that there is not that much communication because I cannot understand him when he is talking. I can barely make out what he is trying to say. I can ask him a question and he will struggle to answer me.’ (Participant 2, 23 years old, daughter)

‘Yes, it’s tough. Sometimes I feel like I am losing patience with her. She keeps saying ‘*leyami*’ [*‘that’s mine’ – a contextually meaningless word in isiZulu*] and I really don’t understand. I just get impatient. Sometimes I even say to her that she must not say anything, because I don’t understand what she really wants. I have lost my patience with her because *ëyi* [*‘hey’ – exclamation*] it’s been what, 8 months and her speech is not back. Like normal, like before the stroke.’ (Participant 1, 21 years old, daughter)

### Support

The lack of support, especially from some family members, resulted in the caregivers struggling to cope with the sudden burden of caring for the PWA. The lack of financial and social support resulted in the feeling of neediness and loneliness, which in turn further exacerbated the burden of caregiving. Financial and social support tended to lessen as time progressed, as illustrated in the quotes from participants 1 and 13 below:

‘Support laughs. The family is alive and well. One of them is in Daveyton and the other lives here in Tembisa. Families don’t say anything about them. They are the same family that never comes anymore. You know what, family is there when you are well and have money. As soon as you get ill, they are gone. Family laughs. One last came in December and the other in January.’ (Participant 13, 53 years old, wife)

‘Ja [*Yes*], support was there. Ja [*Yes*], especially at the time when she had just had her stroke attack. When she had just had her stroke, there was so much support, but now I feel like people are tired or something, I really don’t know. All I know is that we currently don’t have as much support as we had in the beginning.’ (Participant 1, 21 years old, daughter)

### Emotions: Burnout and burden of care

Participants reported that they go through a lot of emotional and psychological difficulties as a result of caregiving. Most of them had to make lifestyle changes to accommodate the extra burden of caring for their family members – a role that was expected of them as spouses, the elder sibling and generally as women. The physical pain experienced, especially by the elderly participants, and the emotional pain of having someone depend on them was too heavy for the caregivers to bear. None of the participants had spoken to anyone about the caregiving experience before this study was conducted. As a result, eight participants were referred to a psychologist for counselling after the interviews.

‘I felt pain. So much pain. You see because it was such a change. Let me tell you something that you probably don’t know about me. I am person of arthritis and all the other things that affect a person at the old age and it therefore becomes very difficult to have to carry this person, think for this person and wash this person whether I like it or not.’ (Participant 14, 65 years old, wife)

‘I felt pain. I felt deep pain, because of this thing of him not talking. Because of this talking thing.’ (Participant 13, 53 years old, wife)

‘I was painful, wena [*‘you’ – exclamation*]. You … you know that person is a breadwinner and everyone in his family is looking up to him. He is supporting everyone. Then it was painful, but I think that I am recovering now.’ (Participant 8, 37 years old, wife)

### Challenges to care

The lack of finances and poverty came across very strongly in the participants’ responses. Because of the lack of finances, participants often sacrificed rehabilitation visits for basic needs such as food for the family. Barriers of access to healthcare such as transportation, the long waiting periods and having to pay multiple times for different services proved to be challenging for the participants. For the older participants, their biggest challenge was that of physically caring for the PWA, such as lifting them during bath time.

‘What I can say is that he was supporting us financially and now he is not working, so things are not good. Now we are being supported by my mother alone. She is now working alone. Now she has to pay my father’s medical bills, pay for my sister’s child at school, run the household and also pay for my tertiary fees. It is very difficult when the source is just only one person. We barely get by monthly.’ (Participant 2, 23 years old, daughter)

‘I don’t think that you bring stroke upon yourself, so I don’t think that it is necessary for us to be paying for everything. I mean in one visit to the hospital, you pay at the doctor’s, at the physios, at OT and at Speech. How much is that all together. Now I have had to stop OT and speech and we can only afford physio for now.’ (Participant 1, 21 years old, daughter)

### Feminisation of caregiving

All of the participants of the study were female primary caregivers. There appears to be a notion among participants that the role of caring is one that should be taken on by women as per cultural norms. Primary caregivers of people with aphasia have had to change their routines, sacrifice the time that they used to make money and also sacrifice their social lives to care for the PWA because it was expected of them as women. This is evident in what the following participants said:

‘We are a family of three boys and two girls, it was therefore decided that being the eldest girl, my sister would be better cared for by me. I run a spaza outside my house, so that I can be near her when she needs me. The money from my shop really helps us with the buying of the food. I have applied for the grant, which I am hoping will be approved this time as we need the money.’ (Participant 11, 40 year old, sister)

‘My father-in-law came to live with us because my husband is the first born. And being the daughter-in-law I have to take care of him. Remember in our culture you marry the whole family. I was working full time, but I have had to take part-time employment so that I can spend some time to care for him. Yoh [*exclamation*], it’s not easy.’ (Participant 4, 28 years old, daughter-in-law)

## Discussion

The study reveals a distressing picture of the hardships that caregivers endure in order to ensure the well-being of the PWA in a context of poverty in South Africa. The current study highlighted how primary caregivers of PWAs had some knowledge about a stroke but did not understand the extent of language and communication impairments associated with aphasia. Caregivers attributed the causes of a stroke to many aspects such as lifestyle aspects, falls, problems with brain and witchcraft. These findings resonate with studies by Penn (2014) and Legg and Penn (2013a) whose findings in Khayelitsha in the Western Cape of South Africa, a large sprawling township characterised by similar levels of poverty and limited resources, were that different people had different understandings of aphasia as well as differing views of aetiology (Legg 2010; Legg & Penn 2013a). The lack of information on aphasia that the PCPWAs had access to in Legg’s research (2010) as well as the current study highlighted their poor health literacy which has been identified in many communities (Sherratt 2011). Even though participants in this study attributed aphasia to a different cause, they continued to seek western ways of intervention such as speech therapy, however there seemed not to have been a holistic management to include caregiver education and strategies to facilitate communication with PWA.

The lack of knowledge about conversational skills on the part of both the caregivers and PWA in the current study resulted in feelings of frustration and humiliation. These findings are mirrored in other international studies, such as those by Avent et al. (2005), Blom Johansson, Calsson and Sonnander (2011) and Blom Johansson (2012). A significant decline in communication, along with changes in everyday life conversations, can further complicate the task of caregiving. Reduced communication in the home environment results in strained relationships that were previously healthy among family members.

The burden of care is placed firmly on the women in this community, which speaks for the feminisation of caregiving, not just in this study but in the African context as well. The impact of caregiving was notable in women, in particular spouses, daughters, daughters-in-law and sisters, who were the primary caregivers in the study. This emphasises the gendered impact of disability care and livelihoods as demonstrated in African and international studies such as those by Zuurmond et al. (2016) and Bakas et al. (2006). The feminisation of caregiving is coupled with high levels of poverty experienced by the women in this study. As in previous studies on caregiving for persons with disabilities in Africa, poverty was often the result of the lack of income from family (Makiwane & Berry 2013) as the PWA was mostly the breadwinner and also because the caregiving role did not allow enough time and opportunities for caregivers to be employed (Cordier 2014; Makiwane & Berry 2013). The participants of the study were women who by virtue of their age should have been still active in the economy but could not do so because of their role as caregivers. This economic marginalisation therefore has implications for gender, social and human rights (Erreguerena 2015).

The lack of social support, especially from family members, further exacerbated the burden of caring for the PWA, as there was no one with whom the caregiver’s role could be shared. Caregivers in this study felt lonely, which is often the case when after the onset of disability, family and social networks are weakened (Oosterhoff & Kett 2014); however, it is during this time that caregivers and family members need to support each other (Howe et al. 2012; Le Dorze & Signori 2010). The lack of social engagement and support also resulted in feelings of isolation and loneliness clearly expressed by the participants, as has been found in other studies (Geiger 2012; Mathye & Eksteen 2015).

The burden and the emotional distress caused by the PWAs not being able to take care of themselves and thus being entirely dependent on the caregivers was evident in this study. The self-care limitations experienced by PWAs resulted in strain experienced by primary caregivers. This was particularly the case with elderly caregivers, because this was an added burden as they already had health concerns of their own. Awareness of their psychological and emotional needs was linked to the level of education of participants. Participants with higher levels of education could recognise that caring for their family members had brought on psychological and emotional problems. There was evidence of deep pain among participants. Most of the pain that was experienced was attributed not only to the change in interactions between the caregivers and the PWA but also to the physical pain that caregivers experience, as a result of having to assist PWA with daily activities that required physical assistance. The pain was as a result of feeling sorry for the PWA and the feeling of losing a breadwinner. Some caregivers however appeared to be more sympathetic towards the pain experienced by the PWAs. The emotional breakdown experienced by some of the participants in the current study when speaking about their concerns was not anticipated at the start of this study, and it is recommended that future studies should take this into consideration.

## Conclusion

The need for knowledge on aphasia, communication strategies and support for caregivers as part of the management of PWA cannot be overly emphasised. Therefore, there is a need to re-evaluate the means of delivering this care, especially in economically disadvantaged areas such as townships. It has been established that factors intrinsically linked with poverty are the ones that make up social determinants of health. Consequently, healthcare, poverty and community development are inseparable. We need to establish partnerships between health, education and the community development sectors, where there are integrated, bottom-up, people-centred, community-based programmes such as community-based rehabilitation. Speech language therapists can liaise with community-based health workers, traditional healers, non-governmental organisations and primary healthcare nurses, who have an understanding of the culture and context. These teams would be used as a vehicle to provide community-based healthcare, reinforce health literacy and provide support and empowerment to PCPWA and PWAs.
